# The Naples pediatric food allergy (NAPFA) score: A multivariable model for the prediction of food allergy in children

**DOI:** 10.1111/pai.70071

**Published:** 2025-03-31

**Authors:** Laura Carucci, Lorenza Biancardi, Rita Nocerino, Letizia Ciliberti, Erika Caldaria, Giorgio Bedogni, Francesco Palmese, Francesco Calabrò, Roberto Berni Canani

**Affiliations:** ^1^ Department of Translational Medical Science University of Naples “Federico II” Naples Italy; ^2^ ImmunoNutritionLab, CEINGE Advanced Biotechnologies Research Center University of Naples “Federico II” Naples Italy; ^3^ Department of Biomedicine and Prevention University of Rome “Tor Vergata” Rome Italy; ^4^ Department of Medical and Surgical Sciences Alma Mater Studiorum‐University of Bologna Bologna Italy; ^5^ Department of Primary Health Care, Internal Medicine Unit Addressed to Frailty and Aging “S. Maria Delle Croci” Hospital, AUSL Romagna Ravenna Italy; ^6^ Department of Mathematics and Applications “Renato Caccioppoli” University of Naples “Federico II” Naples Italy; ^7^ Task Force on Microbiome Studies University of Naples “Federico II” Naples Italy; ^8^ European Laboratory for the Investigation of Food‐Induced Diseases University of Naples “Federico II” Naples Italy

**Keywords:** anaphylaxis, atopy patch tests, food allergy diagnosis, food protein induced enterocolitis syndrome, oral food challenge, serum specific IgE, skin prick tests

## Abstract

**Background:**

Food allergy (FA) is one of the most common chronic conditions in children. Diagnostic delays and errors in FA are relevant problems in clinical practice. Non‐invasive and accessible tools for FA diagnosis are highly required. We aimed to develop an easy‐to‐use clinical score to facilitate the diagnostic approach for pediatric FA (i.e. the NAPFA score).

**Methods:**

Subjects with suspected FA aged 0–14 years were prospectively evaluated at a tertiary center for pediatric allergy, gastroenterology, and nutrition.

Upon completing the diagnostic workup, the subjects were diagnosed with FA based on the oral food challenge result, or with other conditions. Bootstrapped multivariable logistic regression was employed to construct two models that estimate the probability of having FA, one (M1) without the results of the allergy screening tests, while the other (M2) including them.

**Results:**

Six hundred and twenty‐seven pediatric subjects were included in the study. The median (interquartile interval) age at symptom onset was 8 (3;27) months. M1 employed the following predictors: sex, age at symptoms onset, cesarean delivery, occurrence of atopic dermatitis before FA onset, first degree family members with allergy, symptoms occurrence after ingestion of specific food, and skin, gastrointestinal, respiratory, and systemic symptoms. M2 replaced the occurrence of symptoms after ingestion of specific food with the results of allergy tests. The c‐statistic was 0.915 (95% bootstrapped CI: 0.895–0.937) for M1 and 0.977 (95% CI: 0.969–0.992) for M2. Both models demonstrated good internal calibration and a favorable decision analysis curve.

**Conclusion:**

The NAPFA score could be an easy‐to‐use tool holding the potential to streamline the FA diagnostic process in pediatric age, reducing unnecessary testing, and improving patient outcomes in a variety of healthcare settings. Its external validation will possibly enable a standardized approach for identifying children with FA.

**Abbreviations**
APT: atopy patch tests
COMISS: Cow's Milk‐related Symptom Score
DCA: Decision curve analysis
EAACI: European Academy of Allergy & Clinical Immunology
EATERS: Exposure, Allergen, Timing, Environment, Reproducible Symptoms
FA: Food Allergy
FPIES: food protein induced enterocolitis syndrome
M1: nomogram including anamnestic and clinical features
M2: nomogram including anamnestic, clinical features, and result of allergy tests
NAPFA: Naples Pediatric Food Allergy
OFC: oral food challenge
SIAIP: Italian Society of Pediatric Allergy and Immunology
sIgE: specific serum IgE
SIGENP: Italian Society of Pediatric Gastroenterology, Hepatology, and Nutrition
SPT: prick tests
TRIPOD+AI: Transparent Reporting of a multivariable prediction model for Individual Prognosis Or Diagnosis + Artificial Intelligence

**Key message**

The NAPFA score could be an easy‐to‐use tool, combining anamnestic and clinical features to predict the probability of FA diagnosis in children, even without the availability of allergy test results. It can be used by various healthcare professionals, facilitating pediatric FA diagnosis and potentially cutting healthcare costs and waiting lists.

## 1 INTRODUCTION

Food allergy (FA) is one of the most common chronic conditions in children.^1^ The prevalence, incidence, persistence, and severity of FA are on the rise, as recently suggested by epidemiologic studies.^2^, ^3^, ^4^ This epidemiologic pattern is associated with an increase in the economic burden related to the management of pediatric FA, presently estimated to be € 3.820/year/child.^5^ The diagnostic procedures contribute substantially to the financial and psychological burden of pediatric FA, and in many cases, the FA diagnosis remains presumptive or delayed.^5^ The overdiagnosis of FA is a substantial concern, as evidenced by an up to 15‐fold disparity between self‐reported and challenge‐verified prevalence rates in the pediatric age.^6^ Overdiagnosis increases the need for tertiary center access, with a negative impact on the waiting lists. The waiting lists are one of the main criticisms of the National Healthcare System in Italy, as they dramatically compromise accessibility and availability of healthcare services.^7^ Finally, diagnostic delay remains a significant challenge in pediatric FA, particularly for non‐IgE‐mediated FA, in which it is estimated to be up to 6 months. This delay leads to additional psychological and economic burdens for both patients and the healthcare system.^8^, ^9^ The available scores or questionnaires for the diagnosis of pediatric FA are mainly focused only on cow's milk allergy or on IgE‐mediated FA and mostly require the use of allergy tests.^10^, ^11^, ^12^, ^13^, ^14^ Therefore, tools for facilitating the diagnostic approach for pediatric FA without excessive reliance on testing are urgently needed.

The goal of the Naples Pediatric Food Allergy (NAPFA) project was to develop a standardized scoring system that incorporates anamnestic and clinical data, as well as allergy test results, to facilitate the diagnostic process for children with suspected FA.

## 2 METHODS

### 2.1 Study design

Prospective study performed at the Tertiary Care Center for Pediatric Allergy, Gastroenterology, and Nutrition of the Department of Translational Science at the University “Federico II” of Naples. The multidisciplinary team at the Center, comprising pediatricians, allergists, dietitians, and nurses with expertise in pediatric FA, provided a list of the most relevant anamnestic and clinical features associated with these conditions. The list was discussed during three meetings, and predictors for modeling were selected from this list only if ≥80% of team members agreed (see Section 4.2). Items lacking this level of agreement were excluded. Furthermore, conflicting data, data exclusive to a specific type of FA (e.g., IgE vs. non‐IgE), or predictors without a clear association with the occurrence of FA, were also considered for removal. The following factors were excluded from the analysis: living conditions, formula consumption in the first week of life, the timing of symptom onset after consuming specific foods, and antibiotic use. The reporting of the study was performed according to the TRIPOD‐AI+ guidelines, and the TRIPOD‐AI+ checklist is enclosed as File S1.^15^

### 2.2 Ethics

The study was approved by the Ethics Committee of the University “Federico II” of Naples (Protocol number 283/21), registered at www.clinicaltrials.gov as NCT05707858, and conducted in accordance with the Helsinki Declaration (Fortaleza revision, 2013), the Good Clinical Practice Standards (CPMP/ICH/135/95), the Italian Law 211/2003 regarding personal data, and the European regulations on this subject.

### 2.3 Study population

Eligible for the study were Caucasian subjects of both sexes, aged 0–14 years, who were consecutively referred to our Tertiary Center for Pediatric Allergy, Gastroenterology, and Nutrition due to a suspicion of FA raised by their family pediatrician or by other physicians operating in other hospitals. FA was suspected based on a positive history for the following symptoms: (1) skin symptoms (urticaria, angioedema, itching, atopic dermatitis (AD)); (2) gastrointestinal symptoms (vomiting, abdominal pain, constipation, gastroesophageal reflux, bloody stools, diarrhea); (3) respiratory symptoms (nasal itching, sneezing, rhinorrhea, congestion, conjunctivitis, cough, chest tightness, wheezing, shortness of breath); (4) systemic symptoms (irritability, lethargy, marked pallor, hypotension, shock).

Exclusion criteria were age >14 years, presence of chronic systemic diseases, malignancies, immunodeficiencies, infectious diseases, autoimmune diseases, inflammatory bowel diseases, celiac disease, metabolic and genetic diseases, cystic fibrosis, chronic pulmonary diseases, gastrointestinal, respiratory, urinary tract or cardiovascular malformations, neurologic or neuropsychiatric disorders, eosinophilic gastrointestinal disorders.

### 2.4 Study procedures

**FIGURE 1:**
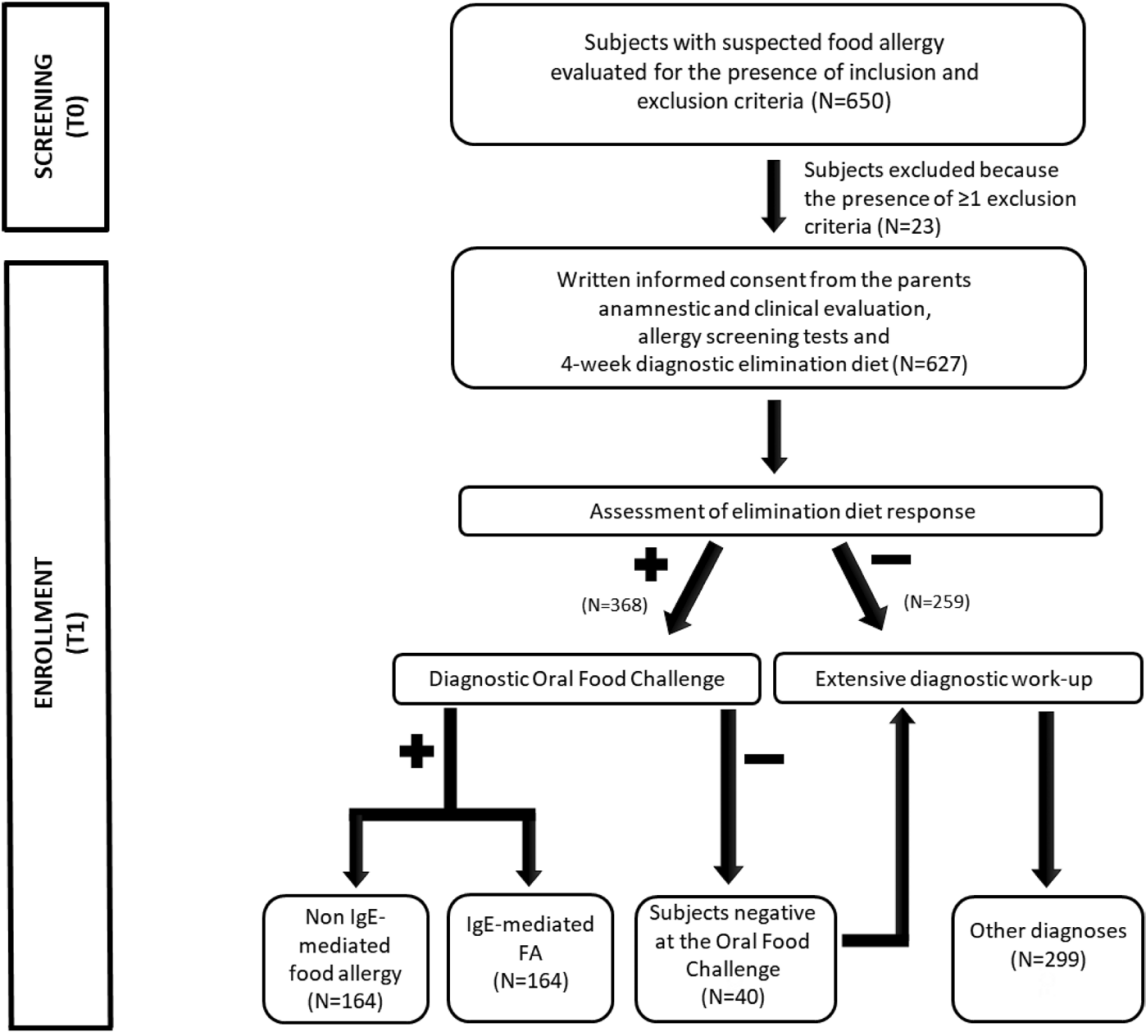
The study design.

The study procedures are depicted in Figure 1.

During the initial visit, the multidisciplinary team operating at the Center performed a complete anamnestic and clinical evaluation to assess the eligibility of subjects, collected the written consent from the parents of each subject, and performed FA screening tests, (skin prick tests, SPT; atopy patch tests, APT; and/or measurement of food‐specific serum IgE, sIgE levels). At the end of the initial visit, certified dietitians provided the parents with written instructions for a four‐week elimination diet, which was tailored to medical history. During the second visit, children who did not respond to the elimination diet were deemed non‐allergic and underwent a comprehensive diagnostic work‐up. In patients who were responsive to the elimination diet and had complete disappearance of FA‐related signs and symptoms, a diagnostic oral food challenge (OFC) was planned. In patients with suspected multiple FA, the OFC was planned with one food at a time. In patients with anaphylaxis or food protein‐induced enterocolitis syndrome (FPIES) induced by the ingestion of a single food, the OFC test was not performed, as suggested by other authors.^16^, ^17^ The OFC was performed at the Hospital within 7 days from the second visit. Children with OFC‐proven FA were categorized as having IgE or non‐IgE FA on the basis of their clinical features.^16^, ^18^, ^19^ Children who were negative at OFC underwent an extensive diagnostic work‐up, and alternative diagnoses were obtained.

### 2.5 Atopy patch tests

The APT were performed based on the clinical history by using fresh foods, as detailed elsewhere.^20^, ^21^ Patients who were taking antihistamines or steroids were advised to stop these medications 7 days before APT.

### 2.6 Skin prick tests

The SPT were performed using extracted allergens and fresh foods based on the clinical history, as described in detail elsewhere.^20^, ^21^ Patients who were taking antihistamines or steroids were advised to stop these medications 7 days before the SPT.

### 2.7 Serum specific IgE levels

Food‐specific serum IgE levels were assessed by enzymatic immunoassay (Phadia 100, ThermoFisher Scientific, Rodano, Milano, Italy). Measurements were expressed as kU/L.

The list of food antigens to test was chosen based on the anamnestic features of each study subject.

### 2.8 Oral food challenge

All OFC were performed at our Tertiary Center for Pediatric Allergy, Gastroenterology, and Nutrition. Patients taking antihistamines or steroids were advised to stop these medications 7 days before the OFC. Peripheral intravenous access was secured before testing. The preparation of the food was conducted by experienced FA dietitians who were not directly involved in the OFC. Patients without a suggestive history of FPIES received protein doses of suspected foods in doses of 3, 10, 30, 100, 300, 1000 and 3000 mg every 20 minutes.^18^ In patients with a history suggestive of FPIES and suspicion of multiple FA, the OFC was done one food at a time with a 48‐h interval. The patients received 0.3 g/kg body weight of the specific food protein in three equal doses over 30 minutes.^16^ The OFC was stopped and considered diagnostic for FA if there were any objective signs of an allergic reaction or if subjective symptoms occurred after the consumption of at least three doses of the tested food, or, in subjects with FPIES, if the major criterion was present together with ≥2 minor criteria.^16^, ^18^ All patients were observed for 6 h after the final dose or for 6 h after symptoms resolution in case of a positive OFC. All essential emergency equipment spediatrics and medications, including epinephrine, antihistamines, steroids, as well as ondansetron and saline solution, were readily available. The OFC results were assessed by the multidisciplinary team operating at the Center. If the patient did not show any signs during the hospital OFC, parents were instructed to give a single feed of the maximum dose administered at the hospital every day for 30 days at home. If the patients had any symptoms during this period, the parents were advised to return to the Center on the same day to have the multidisciplinary team look at the challenge results. After 30 days of home food administration, the patients were re‐examined and their parents interviewed at the center. The OFC was considered negative if the patient tolerated the challenge, including the 30 days of observation.

## 3 STUDY AIM

The aim of the study was to develop and internally validate a clinical scoring system for facilitating the diagnostic approach in pediatric patients with suspected FA.

## 4 DEVELOPMENT AND INTERNAL VALIDATION OF MULTIVARIABLE REGRESSION MODELS

### 4.1 Outcome variable

The outcome of the multivariable regression models was FA, as diagnosed by the OFC.^18^ By our choice, the model did not distinguish between IgE and non‐IgE mediated FA.

### 4.2 Predictor variables

As predictors, we used the most known risk factors for FA and anamnestic and clinical features suggestive of FA, coded as follows:

1. sex (discrete, 0 = female; 1 = male)^22^, ^23^;
2. age at the onset of the signs and symptoms possibly related to FA (continuous, months)^1^, ^22^;
3. cesarean delivery (discrete, 0 = no; 1 = yes)^22^, ^24^;
4. first degree family member with allergy (discrete, 0 = no; 1 = yes)^22^, ^23^;
5. occurrence of atopic dermatitis before the onset of the signs and symptoms possibly related to FA (discrete; 0 = no; 1 = yes)^22^, ^23^;
6. symptoms occurrence after ingestion of specific food (discrete; 0 = none; 1 = 1 time; 2 = ≥2 times)^16^, ^17^;
7. presence of skin symptoms of FA including at least one among urticaria, angioedema, itching, and atopic dermatitis (discrete; 0 = no; 1 = yes)^17^, ^25^;
8. presence of gastrointestinal symptoms including at least one of vomiting, abdominal pain, constipation, gastroesophageal reflux, bloody stools, diarrhea; (discrete; 0 = no; 1 = yes)^17^, ^19^, ^25^;
9. presence of respiratory symptoms including at least one among nasal itching, sneezing, rhinorrhea, congestion, conjunctivitis, cough, chest tightness, wheezing, or shortness of breath; (discrete; 0 = no; 1 = yes)^17^, ^25^;
10. presence of systemic symptoms including at least one among irritability, lethargy, marked pallor, hypotension, and shock; (discrete; 0 = no; 1 = yes)^16^, ^17^, ^19^, ^25^;
11. positivity of SPT, APT, or food‐specific IgE levels (discrete; 0 = no; 1 = yes).^16^, ^17^, ^25^, ^26^

A positive SPT was defined as having a diameter ≥3 mm, a positive IgE as having a value of ≥0.35 KU/L, and a positive APT as having erythema and infiltration after 72 h (48 h of occlusion time).

### 4.3 Sample size estimation

Before performing any modeling, we selected a list of predictors (see Section 4.2) and calculated the frequency of FA among the children. We used the number of predictors^11^ and the frequency of FA (52%) to evaluate the minimum sample size needed to minimize overfitting and allow a precise estimation of model parameters.^27^, ^28^ In detail, we calculated that 627 subjects were needed to detect a Cox‐Snell *R* ^2^ of .377, corresponding to a C‐statistic of 0.81, which we deemed as the minimal acceptable optimism‐corrected discrimination, while ensuring (1) a shrinkage of predictor effects <5%, (2) a difference of 5% in the model apparent and adjusted Nagelkerke *R* ^2^, and(3) estimation within 5% of the average outcome risk in the population.^27^, ^28^, ^29^

### 4.4 Missing data

There were no missing data.

## 5 STATISTICAL ANALYSIS

Most continuous variables were not Gaussian‐distributed, and all are reported as median (50th percentile) and interquartile interval (25th and 75th percentiles). Discrete variables are reported as the number and percentage of subjects with the characteristic of interest. We used logistic regression to develop a multivariable regression model (M1) using FA as outcome and the following predictors: (1) sex, (2) age at the onset of signs and symptoms possibly related to FA, (3) cesarean delivery, (4) occurrence of atopic dermatitis before the onset of signs and symptoms possibly related to FA, (5) first degree family member with allergy, (6) symptoms occurrence after ingestion of specific food, (7) skin symptoms, (8) gastrointestinal symptoms, (9) respiratory symptoms, and (10) systemic symptoms. Because the predictor “symptoms occurrence after ingestion of specific” food had two levels, the number of effective predictors was 11. A further multivariable logistic regression model (M2) was developed, replacing the symptoms after food ingestion with SPT/APT/sIgE results as the two predictors were collinear, resulting in a total of 10 effective predictors. Predictors were kept in the model regardless of whether they were statistically significant.^30^ Overall fit was evaluated using the scaled Brier score, that is, the Brier score scaled by its maximum score (Briermax) according to the equation (1 − Brier score)/Briermax, with a higher score representing greater accuracy.^31^ Discrimination, that is, the ability to separate subjects with disease from those without disease, was evaluated using Harrell's C‐statistic which, for the case of logistic regression, equals the area under the receiver‐operating characteristic curve.^32^ Calibration, that is, the agreement between observed and predicted risk, was assessed by evaluating: (1) “mean calibration” or “calibration‐in‐the‐large” (CITL), by comparing the observed event rate with the average predicted risk; (2) “weak calibration”, by performing a logistic analysis testing whether the calibration slope is 1; and (3) “moderate calibration”, by using a “calibration plot” to test whether the predicted risks correspond to the observed event rates. Such a graph plots the predicted (expected) outcome probabilities (*x*‐axis) against the observed outcome frequencies (*y*‐axis). We used a locally weighted scatterplot estimator with 95% CI to assess how well the model prediction lies around the 45‐degree line of the calibration plot.^15^ All models were internally validated by calculating the scaled Brier score, C‐statistic, CITL, and calibration slope on 1000 bootstrap samples with replacement^29^, ^32^, ^33^, ^34^ and drawing a calibration plot with 95% confidence intervals. The linearity of the logit of age in all models was evaluated using multivariable fractional polynomials with bootstrap evaluation of stability.^35^, ^36^ Age was found to be linear in all models and was modeled as such. Collinearity among predictors was assessed by evaluating the condition matrix^37^ and by using Spearman's rho.^38^ Collinearity was detected between SPT/APT/sIgE results and the appearance of symptoms after the ingestion of specific food so they were not used together in the same model as already reported above (Section 5). Besides giving the regression equations of the models, we developed a nomogram to simplify their use in clinical practice.^39^ Decision curve analysis (DCA) was also employed to evaluate clinical utility, which refers to the implications of model adoption in clinical practice.^40^ Statistical analysis was performed using Stata 18.5 (Stata Corporation, College Station, TX, US) using the *mfpboot*,^36^ *bsvalidation*,^41^ *pmcalplot*,^42^ *pmsampsize*,^43^ *nomolog*,^44^ and *dca* ^45^ community‐contributed commands.

## 6 RESULTS

### 6.1 Study population

From 24 January 2023 to 20 December 2023, a total of 650 subjects were seen at our Center for suspected FA. Twenty‐three subjects were excluded because of the presence of ≥1 exclusion criterion. In detail, 14 had signs or symptoms of infectious diseases; five were aged >14 years; two had neuropsychiatric diseases; one had a previous diagnosis of celiac disease, and one was affected by cystic fibrosis. Thus, 627 subjects were enrolled in the study and underwent the anamnestic and clinical evaluation including SPT, APT, or food‐specific sIgE level measurement and a 4‐week elimination diet. The primary demographic, anamnestic, and clinical characteristics of these Caucasian pediatric subjects from Southern Europe are presented in Table 1.

**TABLE 1:** Anamnestic, demographic, and clinical features of the study population.

	Non‐food allergy	Food allergy	*p*‐Value
Number of enrolled subjects	299	328	
Sex			
Female	146 (48.8%)	138 (42.1%)	.090
Male	153 (51.2%)	190 (57.9%)	
Age at the onset of the signs and symptoms possibly related to food allergy (months)	20 (6;40)	6 (2;12)	<.001
Cesarean delivery			
No	146 (48.8%)	136 (41.5%)	.064
Yes	153 (51.2%)	192 (58.5%)	
Occurrence of atopic dermatitis before the onset of the signs and symptoms possibly related to food allergy			
No	241 (80.6%)	181 (55.2%)	<.001
Yes	58 (19.4%)	147 (44.8%)	
First degree family member with allergy			
No	150 (50.2%)	97 (29.6%)	<.001
Yes	149 (49.8%)	231 (70.4%)	
Symptoms occurrence after ingestion of a specific food			
No	175 (58.5%)	18 (5.5%)	<.001
1 time	90 (30.1%)	95 (29.0%)	
≥ 2 times	34 (11.4%)	215 (65.5%)	
Skin symptoms			
No	156 (52.2%)	119 (36.3%)	<.001
Yes	143 (47.8%)	209 (63.7%)	
Gastrointestinal symptoms			
No	144 (48.2%)	104 (31.7%)	<.001
Yes	155 (51.8%)	224 (68.3%)	
Respiratory symptoms			
No	292 (97.7%)	282 (86.0%)	<.001
Yes	7 (2.3%)	46 (14.0%)	
Systemic symptoms			
No	296 (99.0%)	279 (85.1%)	<.001
Yes	3 (1.0%)	49 (14.9%)	
Positivity of SPT/APT/food‐specific IgE			
No	294 (98.3%)	44 (13.4%)	<.001
Yes	5 (1.7%)	284 (86.6%)	

*Note*: Continuous variables are reported as median (50th percentile) and interquartile interval (IQI, 25th and 75th percentiles). Discrete variables are reported as the number and proportion of subjects with the characteristic of interest. Between‐group comparisons of discrete variables were performed using Pearson's Chi‐square test and those of continuous variables using the Wilcoxon‐Mann–Whitney test.

Abbreviations: APT, atopy patch test; SPT, skin prick test.

In 368 patients, a complete resolution of signs and symptoms, possibly related to FA, was observed after a 4‐week elimination diet (Figure 1). These 368 subjects responsive to the elimination diet underwent the diagnostic OFC, which resulted positive in 328 (89%) of them. The most common food allergens were cow's milk (*n* = 197, 60.1%), hen's egg (*n* = 86, 26.2%), nuts (*n* = 46, 14.0%), legumes except for soy (*n* = 25, 7.6%), fruits (*n* = 22, 6.7%), fish (*n* = 20, 6.1%), soy (*n* = 18, 5.5%), wheat (*n* = 18, 5.5%), peanuts (*n* = 17, 5.2%), meat (*n* = 12, 3.7%), and rice (*n* = 12, 3.7%). (These frequencies do not sum up to 100% because of multiple FA).

The 40 children negative at the diagnostic OFC underwent an extensive diagnostic work‐up and received the following diagnoses: atopic dermatitis not related to FA (*n* = 10), acute urticaria (*n* = 12), functional abdominal pain (*n* = 7), functional vomiting (*n* = 5), celiac disease (*n* = 4), and hereditary angioedema (*n* = 2).

The 259 children unresponsive to the diagnostic elimination diet underwent a comprehensive work‐up to ascertain the underlying diagnosis. In these children, the following diagnoses were obtained: celiac disease (*n* = 30), functional diarrhea (*n* = 32), functional constipation (*n* = 23), functional vomiting (*n* = 14), functional gastroesophageal reflux (*n* = 16), atopic dermatitis not related to FA (*n* = 53), acute urticaria (*n* = 71), functional abdominal pain (*n* = 11), chronic parasitic infection (*n* = 4), food intolerance (*n* = 2), malformations of the gastrointestinal tract (*n* = 2), and early onset inflammatory bowel disease (*n* = 1).

### 6.2 Development, internal validation, and decision curve analysis of the multivariable models

As described in detail under statistical analysis section, two models were developed that included or did not include APT, SPT, and sIgE. Table 2 reports such models, labeled M1 and M2, and their associated metrics of overall fit, calibration, and discrimination as determined by bootstrap on 1000 samples without replacement. The regression equations of such models are given in Appendix 1.

**TABLE 2:** Multivariable logistic regression models.

	Model M1 (without allergy tests)	Model M2 (with allergy tests)
Sex	0.255 [−0.200, 0.710]	0.012 [−0.663, 0.686]
Age at onset (months)	−0.053 [−0.069, ‐0.037]	−0.066 [−0.093, ‐0.039]
Cesarean delivery	0.421 [−0.038, 0.880]	0.938 [0.233, 1.642]
AD before FA	0.482 [−0.022, 0.986]	0.490 [−0.261, 1.241]
First degree family member with allergy	0.372 [−0.111, 0.854]	0.910 [0.184, 1.637]
Symptoms after specific food – 1 time	1.885 [1.260, 2.511]	–
Symptoms after specific food (≥2 times)	3.482 [2.830, 4.133]	–
Skin symptoms	0.875 [0.240, 1.509]	1.238 [0.375, 2.101]
Gastrointestinal symptoms	1.183 [0.556, 1.811]	1.999 [1.072, 2.927]
Respiratory symptoms	2.296 [1.366, 3.225]	1.699 [0.235, 3.164]
Systemic symptoms	1.454 [0.227, 2.682]	2.515 [0.912, 4.118]
Positivity of SPT/APT/sIgE	–	5.998 [4.867, 7.128]
Intercept	−3.205 [−3.434, ‐2.975]	−4.315 [−4.653, ‐3.978]
Brier scaled	52.5	79.0
C‐statistic	0.915 [0.895, 0.937]	0.977 [0.969, 0.988]
Expected:Observed (E:O) ratio	0.995 [0.925, 1.050]	0.992 [0.922, 1.032]
Calibration in the large (CITL)	0.006 [−0.43, 0.260]	0.016 [−0.361, 0.415]
Calibration slope	0.941 [0.798, 1.081]	0.916 [0.715, 1.120]
*N*	627	627

*Note*: Values are logistic regression coefficients with 95% confidence intervals in square brackets for variables from sex to intercept. 95% confidence intervals are given in brackets for measures of discrimination and calibration.

Abbreviations: AD before FA, Occurrence of atopic dermatitis before the onset of the signs and symptoms possibly related to food allergy; Age at the onset, age at onset of the signs and symptoms possibly related to food allergy; APT, atopy patch test; sIgE, food‐specific serum IgE; SPT, skin prick test; Symptoms after food ingestion, Symptoms occurrence after ingestion of a specific food 1 time; Symptoms occurrence after ingestion of particular food ≥2 times.

The discrimination made by M1 (optimism‐corrected c‐statistic = 0.915, 95% CI 0.895–0.937) and M2 (optimism‐corrected c‐statistic = 0.977, 95% CI 0.969–0.992) models was good and larger than the hypothesized one (0.810, see Section 5).

**FIGURE 2:**
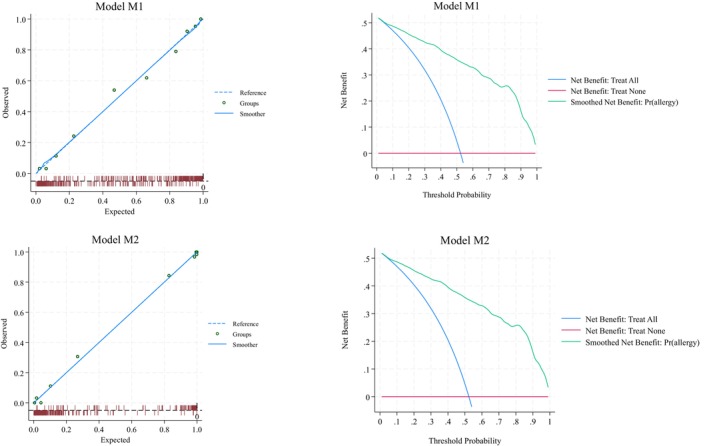
Internal calibration plot and decision curve analysis for food allergy prediction. Calibration plots (left panels) and decision curve analyses (right panels) of the models for the prediction of the probability of food allergy. See Table 2 for the underlying equations.

**FIGURE 3:**
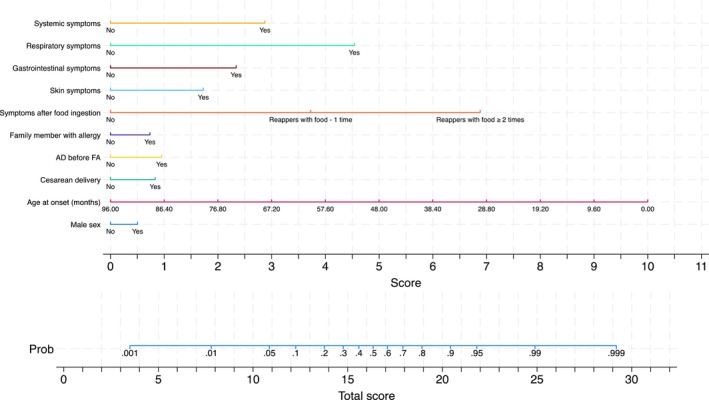
The M1 nomogram. A nomogram that calculates the probability of food allergy in children based on anamnestic and clinical characteristics. See Table 2 for the underlying model. Age at the onset, age at onset of the signs and symptoms possibly related to food allergy; AD before FA, occurrence of atopic dermatitis before the onset of the signs and symptoms possibly related to food allergy; Family member with allergy, first‐degree family member with allergy; Symptoms after food ingestion, symptoms occurrence after ingestion of a specific food 1 time; symptoms occurrence after ingestion of particular food ≥2 times.

**FIGURE 4:**
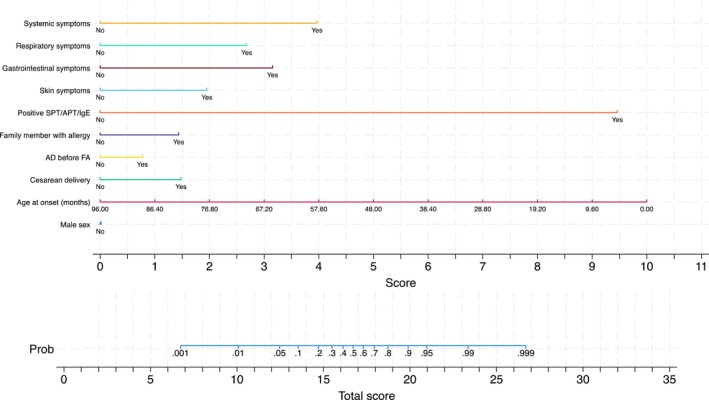
The M2 nomogram. A nomogram that calculates the probability of food allergy in children based on anamnestic and clinical characteristics and results from skin prick test (SPT), allergy panel test (APT), or serum immunoglobulin E (sIgE) levels. See Table 2 for the underlying model. Age at the onset, age at onset of the signs and symptoms possibly related to food allergy; AD before FA, Occurrence of atopic dermatitis before the onset of the signs and symptoms possibly related to food allergy; Family member with allergy, first‐degree family member with allergy; Symptoms after food ingestion, Symptoms occurrence after ingestion of particular food 1 time; Symptoms occurrence after ingestion of particular food ≥2 times. Positive SPT/APT/IgE, skin prick test (SPT) OR atopy patch test (APT) OR food‐specific IgE positivity.

Figure 2 (left quadrants) gives the calibration plots of M1 and M2. At logistic calibration, the average calibration slope was 0.941 for M1 and 0.916 for M2, showing a satisfactory weak calibration. The examination of calibration plots showed an acceptable profile of moderate calibration (Figure 2). We also developed two nomograms corresponding to M1 and M2 (Figures 3 and 4).

Tables S1 and S2 provide the sensitivity, specificity, positive likelihood ratio (LR+), negative likelihood ratio (LR‐), positive predictive value, and negative predictive value for each 10% increment in the probability estimated by Models M1 and M2. LR+ and LR‐ can be useful to estimate post‐test probability from pre‐test probability. Based on LR+ and LR‐, thresholds of 10% and 90% have excellent ability in ruling out and ruling in allergy.

## 7 DISCUSSION

We have developed a scoring system, the NAPFA score, that may help the diagnostic approach in children with suspected FA by providing the probability to be affected by these conditions.

The NAPFA score consists of two multivariable models that can be applied based on the availability of FA screening tests. Its feasibility even without allergy test results allows its application in various healthcare settings, with the potential to reduce overdiagnosis, waiting lists, and associated economic burdens.

Overdiagnosis is a major challenge in pediatric FA that is driven by the reliance on tertiary centers for the confirmation of FA diagnosis, the risks associated with OFC procedures, the psychological stress, and the associated financial costs.^5^ A systematic review conducted in Europe revealed a significant disparity between self‐reported (17.3%) and OFC‐verified (0.9%) FA,^6^ as also observed in our study, where, at the end of the diagnostic process, FA was confirmed in about half of the subjects visiting the center for suspected FA. In addition, diagnostic delay remains a significant problem in pediatric FA. It has a negative impact of disease natural course, and it leads to additional psychological and economic burdens for both families, patients, and healthcare systems.8,9

A tool for facilitating the FA diagnostic approach is urgently needed. Ideally, this diagnostic tool should be able to efficiently address the major limitations that could negatively impact the initial diagnostic approach: definition of the main anamnestic and clinical data that could raise the suspicion of FA; the needs of screening tests to identify the culprit food and of OFC to confirm the diagnosis.

As far as anamnestic data are concerned, we have identified the main variables that have been associated with the occurrence of FA: first‐degree family member with allergies, male sex, born by cesarean delivery, presence of AD before the FA symptoms onset, and young age.^1^, ^16^, ^17^, ^18^, ^19^, ^22^, ^23^, ^24^, ^25^, ^46^ Despite the acknowledged significance of these data, the questionnaires available in the literature primarily focus on clinical symptoms.^10^, ^11^, ^12^, ^13^ Notably, Galvin et al.^10^ were the only authors that developed a model to predict OFC, incorporating sex and age as anamnestic variables. The diagnostic accuracy of Galvin's model was demonstrated by identifying 97% of cases as true positives and 94% as true negatives. However, it was developed specifically for children with IgE‐mediated FA, including only hen's egg, peanuts, or cow's milk allergy. Furthermore, the FA diagnosis was not confirmed by OFC in all study subjects.^10^ The identification of predictors that can be readily gathered in every healthcare setting, such as sex, delivery method, age at onset of signs and symptoms potentially associated with FA, occurrence of AD prior to the onset of signs and symptoms potentially associated with FA, and family allergy risk, makes the NAPFA score an easily and readily accessible tool, but it needs to be verified with further study.

Clinical characteristics are the primary factors determining the suspicion of FA. Clinical features were considered in the Galvin et al. model, which included skin, respiratory, gastrointestinal, and cardiovascular symptoms, but also by the last EAACI guidelines for IgE‐mediated FA; the Exposure, Allergen, Timing, Environment, Reproducible Symptoms (EATERS) method; the COMISS score, and by a Portuguese tool for children with suspected adverse food reactions.^10^, ^11^, ^12^, ^13^, ^14^ To avoid possible errors in evaluating the clinical features, in the NAPFA score we included the signs and symptoms of both IgE‐ and non‐IgE mediated FA, together with the “occurrence of symptoms after ingestion of specific food” as predictors.^25^ The most recent EAACI guidelines on IgE‐mediated FA highlight the relevance of an allergy‐focused history, introducing the concept of a possible OFC‐free FA diagnosis based on the presence of a suggestive clinical history for FA together with SPT or specific sIgE positivity.^11^ The key questions for an allergy‐focused history, as outlined in the EAACI guidelines, are undoubtedly useful in obtaining a focused history for IgE‐mediated FA, and our findings align well with this approach, enhancing the most significant clinical features in children affected by IgE and by non‐IgE‐mediated FA.^11^ In the EAACI guidelines, the time interval between food consumption and clinical symptoms occurrence was considered, as it plays a pivotal role in IgE‐mediated FA, but not in delayed reactions such as non‐IgE‐mediated FA.^25^ Consequently, it was unnecessary to include the timing of symptoms occurrence as a predictor of the NAPFA score, which has been designed for both IgE‐mediated and non‐IgE‐mediated FA. In 2018, a research group developed the EATERS questionnaire. The authors asserted that the presence of several elements of an EATERS history should prompt clinicians to consider the possibility of FA, but the lack of a standardized method for interpreting the questionnaire hindered the comparison with the NAPFA score. In addition, EATERS is not validated yet.^14^ In contrast to the COMISS score, which was initially designed as an awareness tool for cow's milk allergy‐related symptoms^12^ and recently proposed as a diagnostic tool for cow's milk allergy,^47^ the NAPFA score was developed to facilitate the diagnostic approach for children potentially affected by FA caused by any type of food antigens. Furthermore, the NAPFA score used logistic regression to assess the relative contribution of each item while in the COMISS score all items are considered to be equally relevant giving a score ranging from 0 to 6. Consequently, a direct comparison of NAPFA and COMISS is not possible.^12^

Considering the necessity of allergy screening tests to identify the culprit foods, it is important to consider that in certain healthcare settings, FA screening tests may not be readily available. Consequently, without a standardized allergy‐focused medical history, it can be challenging to raise the suspicion of FA, particularly for non‐IgE mediated FA. Notably, the NAPFA score demonstrated remarkable accuracy in diagnosing both IgE‐ and non‐IgE mediated FA, irrespective of the availability of screening tests. Other available models, such as the one proposed by Galvin et al.,^10^ necessitate at least one allergy test between SPT and IgE, rendering it not applicable to non‐IgE mediated FA. This aligns with the EAACI guidelines for IgE‐mediated food allergies.^11^ It appears that the EATERS method stands out as the most effective approach in identifying individuals with IgE‐ and non‐IgE mediated FA without the assistance of FA screening tests. However, as previously mentioned, this method requires standardization and validation.^14^ Furthermore, the COMISS score does not necessitate allergy tests for its applications, but as previously stated, this tool serves as an awareness instrument for cow's milk allergy related symptoms rather than a diagnostic tool. Moreover, its applicability is limited to cow's milk allergy related symptoms, unlike the NAPFA score, which is applicable to a broader range of FA.^12^, ^47^

Finally, while a positive OFC remains the gold standard for the diagnosis of FA; in some contexts OFC, it is not mandatory for FA diagnosis, as in the case of FPIES, anaphylaxis, or for typical IgE‐mediated FA.^11^, ^16^ For the latter case, the combination of a positive allergy test with a suggestive clinical history could be sufficient to perform a diagnosis of IgE‐mediated FA, as stated in the most recent EAACI guidelines.^11^ However, OFC remains essential for the diagnosis of all other FA types.

The NAPFA score demonstrated satisfactory discrimination and calibration and exhibited clinical utility at DCA, potentially facilitating the diagnostic work‐up for all types of FA. However, external validation is necessary to assess its role in clinical practice. As calibration is concerned, the internal validation of our models, performed with bootstrap,^34^ revealed a mean calibration slope of 0.941 (95%CI 0.798 to 1.081) for Model M1 and 0.916 (95%CI 0.715 to 1.120) for Model 2. Consequently, higher predicted probabilities tend to overestimate the risk of FA, while lower predicted probabilities tend to underestimate it. The external validation of the proposed prediction algorithms will allow a potential benefit recalibration, which is a better benchmark of validity as compared to internal validation.^34^

NAPFA may facilitate the early identification of FA children in primary care settings, emergency departments, and tertiary care facilities. It has the potential to effectively improve the circular continuum between these healthcare figures, as recently reported in the Italian Diagnostic Therapeutic Care Pathway (DTCP) for the management of pediatric FA.^48^ The enhancement of this pathway could have a substantial impact on the FA diagnostic process, reducing diagnostic delays, errors, and FA‐related costs.

The NAPFA score's strengths include prospective study design, rigorously determined sample size, well‐defined predictors, and applicability to IgE and non‐IgE‐mediated FA caused by any type of food antigens. Limitations include age restriction to <14 years, ethnicity because the inclusion of Caucasian subjects from Southern Europe only, and the need for external validation. As the latter point is concerned, we plan to perform a multicenter validation study involving several Italian centers in collaboration with the Italian Society of Pediatric Allergy and Immunology (SIAIP) and the Italian Society of Pediatric Gastroenterology, Hepatology, and Nutrition (SIGENP).

## 8 CONCLUSION

The NAPFA score is the first scoring system to incorporate anamnestic and clinical features that facilitate the diagnostic approach of pediatric FA. It is an easily accessible score with good discrimination, calibration, and clinical utility, making it suitable for widespread use by healthcare professionals. Notably, its accuracy and feasibility even without allergy test results enable its application in various healthcare settings, with the potential to reduce healthcare costs and wait times, once externally validated. To facilitate and to speed‐up the external validation of the NAPFA score, also across different populations and settings, we are developing a web app that will enhance the model's accessibility and implementation in clinical practice by easily running NAPFA on computers and mobile devices of interested practitioners.

## AUTHOR CONTRIBUTIONS

**Laura Carucci:** Conceptualization; writing – original draft; investigation; methodology; data curation; visualization. **Lorenza Biancardi:** Investigation; writing – original draft; writing – review and editing. **Rita Nocerino:** Investigation; writing – original draft; writing – review and editing; resources. **Letizia Ciliberti:** Investigation; writing – original draft; writing – review and editing. **Erika Caldaria:** Investigation; writing – original draft; writing – review and editing. **Giorgio Bedogni:** Formal analysis; software; validation; writing – original draft; data curation; resources. **Francesco Palmese:** Formal analysis; software; writing – original draft; resources. **Francesco Calabrò:** Formal analysis; software; writing – original draft; resources; data curation; writing – review and editing. **Roberto Berni Canani:** Methodology; writing – review and editing; writing – original draft; conceptualization; project administration; supervision; data curation; visualization; investigation.

## FUNDING INFORMATION

This research was funded under the National Recovery and Resilience Plan (NRRP), Mission 4 Component 2 Investment 1.3—Call for tender No. 341 of 15 March 2022 of Italian Ministry of University and Research funded by the European Union—NextGenerationEU. Project code PE00000003, Concession Decree No. 1550 of 11 October 2022, adopted by the Italian Ministry of University and Research, CUP E63C22002030007, Project title “ON Foods—Research and innovation network on food and nutrition Sustainability, Safety and Security—Working ON Foods” and from the Italian Ministry of Health‐Health Operational Plan Trajectory 5‐Line of action “Creation of an action program for the fight against malnutrition in all its forms and for the dissemination of the principles of the Mediterranean diet” (Mediterranean Diet for Human Health Lab, “MeDiHealthLab”, code T5‐AN‐07, CUP E63C22002570006).

## CONFLICT OF INTEREST STATEMENT

The authors have no conflict of interest to declare.

### PEER REVIEW

The peer review history for this article is available at https://www.webofscience.com/api/gateway/wos/peer‐review/10.1111/pai.70071.

## Supporting information


Table S1.

File S1.

## Data Availability

Data and code are available upon reasonable request to the corresponding author.

## APPENDIX 1 PREDICTION EQUATIONS

**Table:**

A. Calculate the linear predictor model (LP) as reported in Table 2	LP1 = 0.255*male − 0.053*age_onset + 0.421*cesarean + 0.482*ad_before_fa + 0.372*fam_allergy + 1.885*reappears _1 + 3.482*reappears_2 + 0.875*skin + 1.183*gi + 2.296*resp + 1.454*system − 3.205 or LP2= 0.012*male − 0.066*age_onset + 0.938*cesarean + 0.490*ad_before_fa + 0.910*fam_allergy + 1.238*skin + 1.999*gi + 1.699*lung + 2.515*system + 5.998*SPT_APT_IgE − 4.31 where male = male gender (1 = yes; 0 = no); age_onset = age at the onset of the signs and symptoms possibly related to food allergy (months); cesarean = cesarean delivery (1 = yes; 0 = no); ad_before_fa = Occurrence of atopic dermatitis before the onset of the signs and symptoms possibly related to food allergy (1 = yes; 0 = no); fam_allergy = First degree family member with allergy (1 = yes; 0 = no); reappers_1 = Symptoms occurrence after ingestion of a specific food 1 time (1= yes; 0 =no); reappers_2 = Symptoms occurrence after ingestion of a specific food ≥ 2 times (1= yes; 0 =no); skin = skin symptoms (1= yes; 0 =no); gi = gastrointestinal symptoms (1= yes; 0 =no); resp = respiratory symptoms (1= yes; 0 =no); systemic = systemic symptoms (1= yes; 0 =no) SPT_APT_IgE = skin prick test (SPT) OR atopy patch test (APT) OR food‐specific IgE positivity: 0 = no; 1 = yes
B. Calculate the probability of 1‐year readmission from the linear predictor	Probability = e^LP^ / 1 + e^LP^ where *e* is the base of natural logarithms The probability ranges from 0 to 1. If you wish to have it on a percentage scale, multiply it by 100
