# Supplementary material for: The Naples pediatric food allergy (NAPFA) score: A multivariable model for the prediction of food allergy in children
Source: Pediatr Allergy Immunol. 2025 Mar 31;36(4):e70071. doi: 10.1111/pai.70071 (PMC11956138; doi:10.1111/pai.70071)
Supplement: Supplementary file 1 — Table S1. [file PAI-36-e70071-s001.docx]

**Supplementary Table 1** – Sensitivity (SN), specificity (SP), positive likelihood ratio (LR+), negative likelihood ratio (LR-), positive predictive value (PV+) and negative predictive value (PV-) at different values of the probability estimated by Model M1. Values are point estimates with 95% confidence intervals.

| Probability*100 | SN | SP | LR+ | LR- | PV+ ^§^ | PV- ^§^ |
| --- | --- | --- | --- | --- | --- | --- |
| ≥ 10 | 98.8%  96.9%  99.7% | 72.9%  67.5%  77.9% | 3.65  3.03  4.39 | .0167  .0063  .0444 | 80.0%  75.8%  83.8% | 98.2%  95.5%  99.5% |
| ≥ 20 | 95.7%  92.9%  97.6% | 87.0%  82.6%  90.6% | 7.34  5.47  9.84 | .0491  .0293  .0821 | 89.0%  85.2%  92.0% | 94.9%  91.6%  97.2% |
| ≥ 30 | 95.1%  92.2%  97.2% | 91.0%  87.1%  94.0% | 10.5  7.34  15.1 | .0536  .0332  .0866 | 92.0%  88.6%  94.7% | 94.4%  91.1%  96.8% |
| ≥ 40 | 92.1%  88.6%  94.8% | 95.3%  92.3%  97.4% | 19.7  11.8  32.8 | .0832  .0575  .120 | 95.6%  92.7%  97.6% | 91.6%  88.0%  94.5% |
| ≥ 50 | 91.5%  87.9%  94.3% | 96.7%  93.9%  98.4% | 27.3  14.9  50.3 | .0883  .0619  .126 | 96.8%  94.1%  98.4% | 91.2%  87.5%  94.1% |
| ≥ 60 | 89.9%  86.2%  93.0% | 97.0%  94.4%  98.6% | 29.9  15.7  56.9 | .104  .075  .143 | 97.0%  94.5%  98.6% | 89.8%  86.0%  92.9% |
| ≥ 70 | 89.0%  85.1%  92.2% | 97.7%  95.2%  99.1% | 38.0  18.3  79.1 | .112  .0825  .153 | 97.7%  95.2%  99.1% | 89.0%  85.1%  92.2% |
| ≥ 80 | 86.9%  82.7%  90.3% | 98.3%  96.1%  99.5% | 52.0  21.8  124 | .133  .101  .176 | 98.3%  96.0%  99.4% | 87.2%  83.2%  90.6% |
| ≥ 90 | 85.1%  80.7%  88.7% | 98.3%  96.1%  99.5% | 50.9  21.3  121 | .152  .117  .197 | 98.2%  95.9%  99.4% | 85.7%  81.6%  89.2% |

^§^ Prevalence is 52.0% (48.0% to 56.3%).

**Supplementary Table 2** – Sensitivity (SN), specificity (SP), positive likelihood ratio (LR+), negative likelihood ratio (LR-), positive predictive value (PV+) and negative predictive value (PV-) at different values of the probability estimated by Model M2. Values are point estimates with 95% confidence intervals.

| Probability*100 | SN | SP | LR+ | LR- | +PV ^§^ | -PV ^§^ |
| --- | --- | --- | --- | --- | --- | --- |
| ≥ 10 | 98.8%  96.9%  99.7% | 72.9%  67.5%  77.9% | 3.65  3.03  4.39 | .0167  .0063  .0444 | 80.0%  75.8%  83.8% | 98.2%  95.5%  99.5% |
| ≥ 20 | 95.7%  92.9%  97.6% | 87.0%  82.6%  90.6% | 7.34  5.47  9.84 | .0491  .0293  .0821 | 89.0%  85.2%  92.0% | 94.9%  91.6%  97.2% |
| ≥ 30 | 95.1%  92.2%  97.2% | 91.0%  87.1%  94.0% | 10.5  7.34  15.1 | .0536  .0332  .0866 | 92.0%  88.6%  94.7% | 94.4%  91.1%  96.8% |
| ≥ 40 | 92.1%  88.6%  94.8% | 95.3%  92.3%  97.4% | 19.7  11.8  32.8 | .0832  .0575  .120 | 95.6%  92.7%  97.6% | 91.6%  88.0%  94.5% |
| ≥ 50 | 91.5%  87.9%  94.3% | 96.7%  93.9%  98.4% | 27.3  14.9  50.3 | .0883  .0619  .126 | 96.8%  94.1%  98.4% | 91.2%  87.5%  94.1% |
| ≥ 60 | 89.9%  86.2%  93.0% | 97.0%  94.4%  98.6% | 29.9  15.7  56.9 | .104  .075  .143 | 97.0%  94.5%  98.6% | 89.8%  86.0%  92.9% |
| ≥ 70 | 89.0%  85.1%  92.2% | 97.7%  95.2%  99.1% | 38  18.3  79.1 | .112  .0825  .153 | 97.7%  95.2%  99.1% | 89.0%  85.1%  92.2% |
| ≥ 80 | 86.9%  82.7%  90.3% | 98.3%  96.1%  99.5% | 52  21.8  124 | 133  .101  .176 | 98.3%  96.0%  99.4% | 87.2%  83.2%  90.6% |
| ≥ 90 | 85.1%  80.7%  88.7% | 98.3%  96.1%  99.5% | 50.9  21.3  121 | .152  .117  .197 | 98.2%  95.9%  99.4% | 85.7%  81.6%  89.2% |

^§^ Prevalence is 52.0% (48.0% to 56.3%).
